# Determination of the Crystal Structure and Active Residues of FabV, the Enoyl-ACP Reductase from *Xanthomonas oryzae*

**DOI:** 10.1371/journal.pone.0026743

**Published:** 2011-10-21

**Authors:** He Li, Xiaoli Zhang, Lijun Bi, Jin He, Tao Jiang

**Affiliations:** 1 National Laboratory of Biomacromolecules, Institute of Biophysics, Chinese Academy of Sciences, Beijing, People's Republic of China; 2 State Key Laboratory of Agricultural Microbiology, Huazhong Agricultural University, Wuhan, Hubei, People's Republic of China; National Institute for Medical Research, Medical Research Council, United Kingdom

## Abstract

**Background:**

Enoyl-ACP reductase (ENR) catalyses the last reduction reaction in the fatty acid elongation cycle in bacteria and is a good antimicrobial target candidate. FabV is the most recently discovered class of ENR, but we lack information about the atomic structure and the key residues involved in reductase activity except for the known conserved tyrosine and lysine residues in the Y-X_8_-K active site motif.

**Methodology/Principal Findings:**

Here we report the crystal structure of FabV from *Xanthomonas oryzae* (xoFabV). The crystal structure of this enzyme has been solved to 1.6 Å resolution in space group *P*2_1_2_1_2_1_. The model of xoFabV consists of one monomer in the asymmetric unit which is composed of 13 α-helices and 11 β-strands, representing a canonical Rossmann fold architecture. Structural comparison presents that the locations of the conserved tyrosine (Y236) and lysine (K245) residues in the Y-X_8_-K active site motif of xoFabV and the Y-X_6_-K motif of ecFabI are notably similar. However, the conformations of Y236 in xoFabV and Y156 in ecFabI are distinct. Structure-based site-directed mutagenesis and enzymatic activity assays reveal that in addition to the conserved Y236 and K245 in the Y-X_8_-K motif, Y53, D111 and Y226 are key residues implicated in the reductase activity, and F113 and T276 are also important for enzyme function. Moreover, a proposed active lysine located immediately after the Y-X_8_-K motif in FabV from *Burkholderia mallei* (bmFabV) is altered to an inactive V246 in xoFabV.

**Conclusions/Significance:**

We determine the first crystal structure of the FabV enzyme and identify several residues important for its enzymatic activity. These findings lay a solid foundation for the development of specific antibacterial inhibitors of the pathogenic bacteria, such as *Vibrio cholerae*, *Burkholderia* species and *Xanthomonas* species.

## Introduction

Fatty acid synthesis (FAS) is critical for many organisms except the archaea. The cytosol of mammalian cells and fungi utilise the FAS I system in which the active sites reside in one and two multifunctional proteins, respectively. The FAS II system in bacteria (and in plants, apicomplexan protozoa and mitochondria) uses several discrete monofunctional enzymes that catalyse each single reaction, which makes these enzymes attractive targets for specific antibacterial agents [1], [2]. In particular, enoyl-ACP reductase, which catalyses the last reduction reaction in the fatty acid elongation cycle using *trans*-2-acyl-ACP as the substrate in an NAD(P)H-dependent manner, is a vital enzyme and, therefore, is a promising antimicrobial target. At present, four distinct ENR members, FabI, FabL, FabK and FabV, have been identified. FabI is a member of the short-chain dehydrogenase/reductase (SDR) superfamily, and its coenzyme-binding site has the classical Rossmann fold motif. The homologues from *Escherichia coli* (ecFabI) and *Mycobacterium tuberculosis* (InhA) both have the Y-X_6_-K motif in which the conserved tyrosine protonates the substrate enoyl-thioester and the lysine interacts with the hydroxyl groups of the nicotinamide ribose moiety [1]. FabIs from various bacteria can be inhibited effectively by triclosan, isoniazid or diazaborine through the formation of a FabI-NAD(H)-inhibitor ternary complex [3]–[7]. The interaction between FabI and NAD^+^ is weak [3]; however, FabI can bind strongly to NAD^+^ in the presence of diazaborine or triclosan [3], [6]. Like FabI, FabL from *Bacillus subtilis* (bsFabL) is a member of the SDR superfamily and possesses a Rossmann fold structure and the Y-X_6_-K motif [8]. It is reversibly inhibited by triclosan but does not form a stable ternary complex with NAD^+^ and triclosan [9]. Interestingly, FabK is unrelated to the SDR family. It is a triclosan-resistant flavoprotein [10] that forms a triose phosphate isomerase (TIM) barrel structure [11] rather than the Rossmann fold structure.

FabV is the most newly discovered class of ENR [2]. It exists in a variety of organisms, including some clinically important pathogens and serious plant pathogens, such as *Vibrio cholerae*, *Yersinia pestis*, *Pseudomonas aeruginosa*, *Burkholderia* species and *Xanthomonas* species. FabV from *V. cholerae* (vcFabV) consists of 402 residues and exists as a monomer protein in solution. It is active with both crotonyl-ACP and the model substrate crotonyl-CoA; in addition, FabV strongly prefers NADH to NADPH, and it could not be inhibited effectively by triclosan, even in the presence of NAD^+^. Through the kinetic and mutagenesis studies of FabV from *Burkholderia mallei* (bmFabV) [12], two conserved active site residues, tyrosine (Y235) and lysine (K244), were found to be organised in a Y-X_8_-K motif. Furthermore, a second conserved lysine (K245) residue was identified. Although another conserved tyrosine (Y225) was indicated, no further information was given. Finally, a hydrogen bonding network among these three active residues (Y235, K244 and K245), the cofactor and the enoyl-ACP substrate was proposed. In the hypothetical model, Y235 stabilises the enoyl-ACP substrate and interacts with K244 via a hydrogen bond between the hydroxyl group of Y235 and the side chain nitrogen atom of K244; K244 interacts with both the cofactor NADH and the substrate, while K245 only interacts with the substrate.

Up to now, no crystal structure of any FabV enzyme has been determined. Here, we report the whole structure of xoFabV from *X. oryzae*, a serious pathogen that causes blight disease in rice [13], and we identify the key active residues involved in the reductase activity.

## Results and Discussion

### Crystal structure of xoFabV

The native xoFabV consists of 402 residues, excluding the N-terminal His tag and thrombin cleavage site, with a molecular mass of 45,800 Da. We successfully crystallised the full-length selenomethionine-substituted xoFabV (SeMet-xoFabV) without removing the N-terminal His tag and the thrombin cleavage site. The crystals diffracted to 1.6 Å resolution under synchrotron illumination (Table 1). Phase information was obtained by two-wavelength MAD phasing.

**Table 1:** X-ray diffraction data collection and refinement statistics.

Data collection				
		Se Peak		Se Edge
Wavelength (Å)		0.97902		0.97930
Resolution range (Å)		20−1.60 (1.66−1.60)^a^		20−1.60 (1.66−1.60)
Unique reflections		54,290 (5,366)		54,322 (5,380)
Completeness (%)		99.9 (100.0)		99.9 (100.0)
Redundancy		14.3 (14.6)		14.3 (14.6)
R_merge_ (%)		7.0 (20.5)		6.2 (20.9)
<I/σ(I)>		64.0 (16.4)		63.5 (15.6)
Solvent (%)		44.2%		44.2%
Space group		*P*2_1_2_1_2_1_		*P*2_1_2_1_2_1_
Unit cell (Å)		a = 50.52		a = 50.52
		b = 74.52		b = 74.52
		c = 107.39		c = 107.39
**Refinement statistics**				
Resolution range (Å)			19.89−1.60 (1.66−1.60)	
Residues/ASU^b^			390	
Water molecules/ASU			277	
*R*_work_ (%)^c^			18.5 (14.7)	
*R*_free_ (%)^d^			20.5 (18.7)	
RMSD bond length (Å)			0.006	
RMSD bond angle (^o^)			1.009	
Average B-factors (Å^2^)			14.4	
	Protein		14.1	
	Water molecules		18.0	
Ramachandran Plot				
	In most favoured regions (%)		93.2	
	In additional allowed regions (%)		6.8	
	In generously allowed regions (%)		0	
	In disallowed regions (%)		0	

a Values in parentheses are for the highest resolution shell.

b ASU, asymmetric unit.

c *R*_work_ = Σ||*F*_obs_|–|*F*_calc_||/Σ|*F*_obs_|, where *F*_calc_ and *F*_obs_ are the calculated and observed structure factor amplitudes, respectively.

d *R*_free_ = as for *R*_work_, but for 3.7% of the total reflections chosen at random and omitted from refinement.

**Figure 1:**
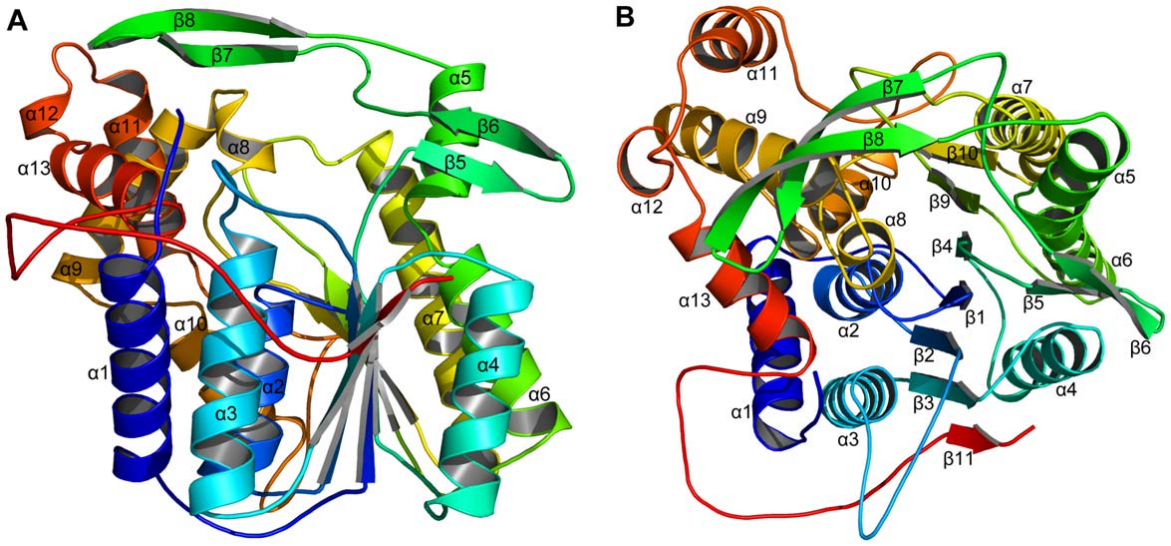
Crystal structure of xoFabV. The structure consists of 13 α-helices and 11 β-strands, representing a classic Rossmann fold architecture. The secondary structures are shown in different colours and are labelled with the corresponding numbers. (A) Side view. (B) Top view. Figures 1–4 were made using PyMOL (DeLano Scientific, Palo Alto, California, USA; http://www.pymol.org).

The SeMet-xoFabV crystal contains one monomer in the asymmetric unit. The density map was of high quality and allowed us to build an almost complete model containing 390 residues (C12-L401) but lacking the His tag and thrombin cleavage site at the N-terminus and A402. Each xoFabV monomer consists of 13 α-helices (α1 – α13) and 11 β-strands (β1 – β11) (Fig. 1). Strands β1 – β4 and β9 – 11 form a parallel β-sheet located at the centre of the structure. This β-sheet is flanked on one side by helices α2, α3 and α10 and on the other by helices α4 – α7, which, taken together, constitute the canonical Rossmann fold centre. The other helices and strands are located around the fold centre. Unlike the strands constituting the β-sheet, strands β5 – β6 and β7 – β8 form two anti-parallel hairpin structures.

### Structural comparison of xoFabV and ecFabI

**Figure 2 pone-0026743-g002:**
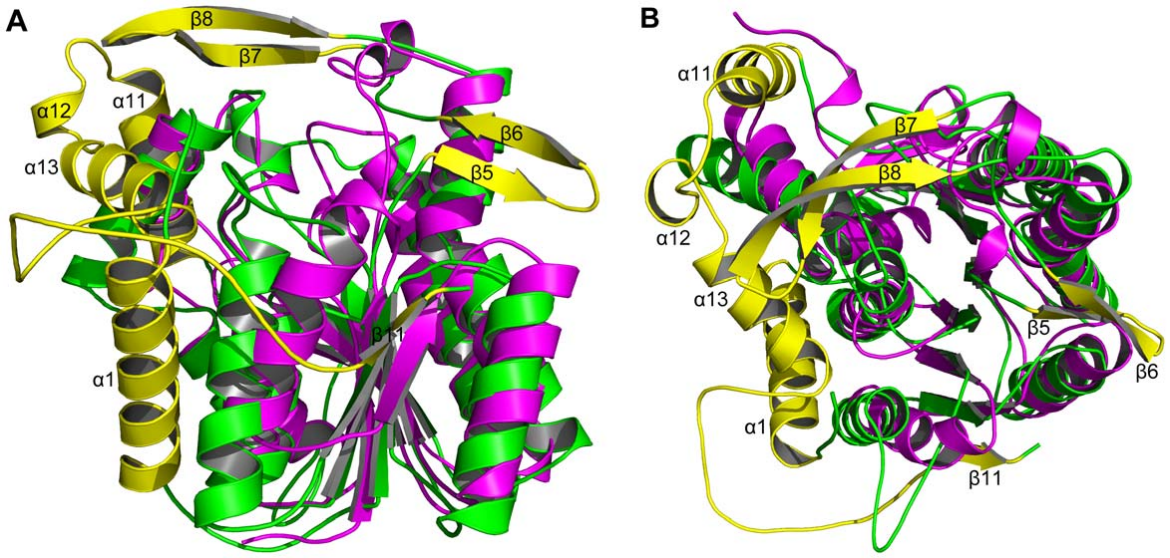
Overall structural comparison of xoFabV and ecFabI. The two structures differ dramatically. The β-sheet and the flanking α-helices are shifted, and xoFabV has additional secondary structures, including helices α1 and α11 – α13 and strands β5 – β8 and β11, which are shown in yellow. Other parts of xoFabV are shown in green; ecFabI is shown in magenta. (A) Side view. (B) Top view.

To find the potential catalytic residues implicated in the reductase activity of xoFabV and to compare the structural differences between xoFabV and its homologue, the structures of xoFabV and chain A of ecFabI (PDB id: 1MFP, ecFabI complexed with NAD^+^ and the inhibitor SB611113 [14]) were superposed using the SSM Superpose function [15] of COOT [16]. Because ecFabI is the most researched homologue of the ENR superfamily and many ternary structures of ecFabI have been resolved [7], [17], [18], 1MFP was chosen as a representative for the structural comparison. Although both xoFabV and ecFabI have Rossmann fold architectures, their overall structures differ dramatically (Fig. 2). The β-sheet and its flanking α-helices constituting the fold centre are shifted, and xoFabV is considerably larger (402 residues) than ecFabI (262 residues) and has additional secondary structures, including helices α1 and α11 – α13 and strands β5 – β8 and β11. However, the functions of these additional secondary structures in xoFabV are still unknown.

**Figure 3 pone-0026743-g003:**
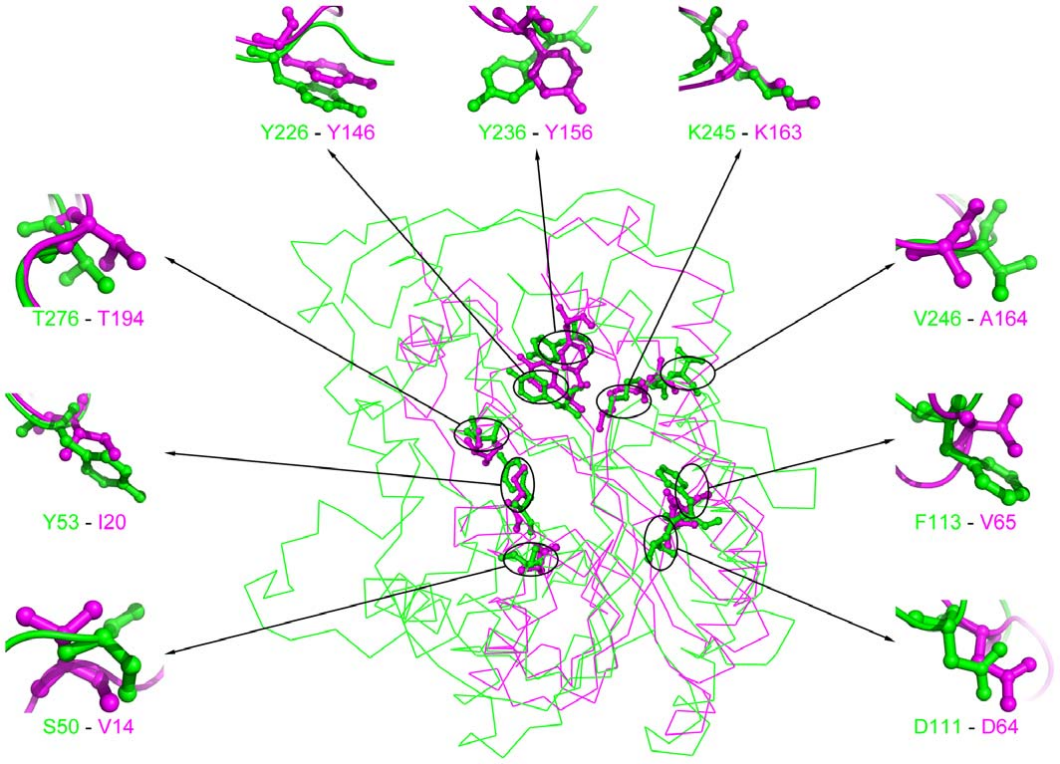
Structural comparison of individual residues in xoFabV and ecFabI. The individual residues listed in Table 2 are shown in sticks and balls. Their positions in the overall structures are labelled with circles and are enlarged. The residues of xoFabV are shown in green and are listed before the hyphen; the residues of ecFabI are shown in magenta and after the hyphen.

Although there was obvious conformational variation between the overall structures of xoFabV and ecFabI, potential active site residues of xoFabV were selected via sequence alignment for detailed conformational comparison (Table 2). First, we selected the residues of ecFabI that are involved in interaction with NAD^+^ as indicated by LIGPLOT analysis [19]. These residues include I20, D64, V65, Y146, K163 (lysine in the Y-X_6_-K motif) and T194 of ecFabI, and the corresponding residues in xoFabV, Y53, D111, F113, Y226, K245 and T276, respectively. Second, we selected Y156, the conserved tyrosine in the Y-X_6_-K motif of ecFabI that interacts with the inhibitor SB611113 via hydrogen bonding and the corresponding residue in xoFabV, Y236 (Fig. 3).

**Table 2:** Residues under comparison.

xoFabV^a^	S50	Y53	D111	F113	Y226	Y236	K245	V246	T276
ecFabI^b^	V14	I20	D64	V65	Y146	Y156	K163	A164	T194

a S50 and Y53 are the predicted conserved residues in the potential NAD(P)H-binding site; V246 is the corresponding residue of K245 in bmFabV; Y236 and K245 are the conserved residues in the Y-X_8_-K motif.

b I20, D64, V65, Y146, K163 and T194 are involved in the interaction with NAD^+^; Y156 interacts with the inhibitor SB611113 via a hydrogen bond. Y156 and K163 are the conserved residues in the Y-X_6_-K motif.

**Figure 4:**
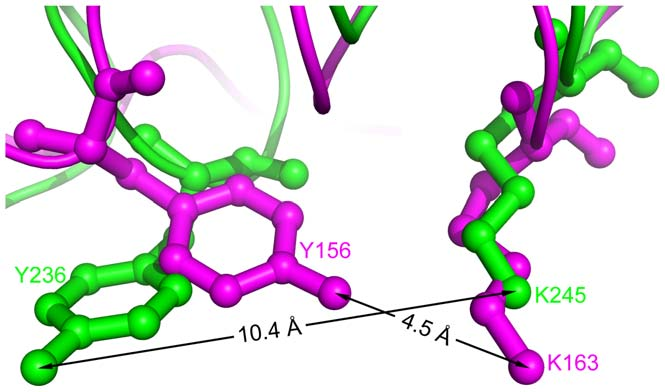
Structural comparison of the Y-X_8_-K motif of xoFabV and the Y-X_6_-K motif of ecFabI. Despite the fact that the Y-X_8_-K motif of xoFabV has two more residues than the Y-X_6_-K motif of ecFabI, the conformations of the conserved tyrosine and lysine residues are similar. The distance between the conserved tyrosine (Y236) and lysine (K245) residues in the Y-X_8_-K motif of xoFabV (shown in green) is 10.4 Å, while the distance between Y156 and K163 in the Y-X_6_-K motif of ecFabI (magenta) is 4.5 Å.

Structural comparison showed that the locations of the conserved tyrosine and lysine residues in the Y-X_8_-K motif of xoFabV and the Y-X_6_-K motif of ecFabI were notably similar, despite the fact that there are two more residues in the former than the latter. However, the conformations of Y236 of xoFabV and Y156 of ecFabI are different (Fig. 4). The side chains of the two tyrosines point to different directions. In ecFabI, Y156 points to K163, and the distance between the oxygen atom of the hydroxyl group in Y156 and the nitrogen atom of the K163 side chain is about 4.5 Å. In xoFabV, Y236 does not point to K245, and the distance is 10.4 Å. The conformations of K163 of ecFabI and K245 of xoFabV are almost identical; they are in the same position, and their side chains point in the same direction. This could be explained by the fact that they are both in a long helix (K245 of xoFabV in helix α7) and are not flexible enough to change conformation. The difference between the distances of the conserved tyrosine and lysine residues might lead to the variations in their abilities to bind the substrate, cofactor or inhibitor. As for the other residues being compared, D111, Y226 and T276 of xoFabV are conserved in ecFabI, and the residues corresponding to Y53 and F113 of xoFabV have been substituted with different amino acids in ecFabI.

**Figure 5:**
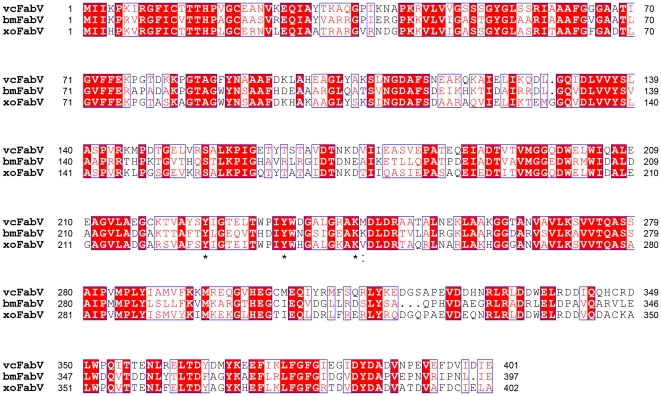
Full-length sequence alignment of three enoyl-ACP reductase enzymes from different organisms. The sequences are from *V. cholerae, B. mallei* and *X. oryzae*. Y226, Y236 and K245 of xoFabV and their corresponding residues in the other two enzymes are labelled with asterisks. V246 of xoFabV and its corresponding residues are labelled with a colon. The sequence alignment was performed using T-Coffee, and the figure was made using ESPript [34].

Although no FabV structure has been reported, previous bioinformatic and mutagenesis studies of bmFabV suggested that in addition to the conserved tyrosine (Y235) and the lysine (K244) residues of the Y-X_8_-K motif, an adjacent lysine (K245) in bmFabV also plays an important role in substrate binding. However, a sequence alignment of vcFabV (YP_001217283.2), bmFabV (YP_102617.1) and xoFabV using T-Coffee [20], [21] revealed that K245 present in bmFabV (Fig. 5) is altered to a non-polar valine (V246) in xoFabV.

In addition, we used CD-Search [22] against the Conserved Domain Database [23] to probe additional potentially key residues; the results showed that S50 and Y53 (previously mentioned in the structural comparison) might be involved in NADH binding.

### Plasmid complementation test and NADH oxidation assay

To determine the interaction network between xoFabV, the cofactor and the inhibitor, we attempted to co-crystallise xoFabV with NAD^+^ and NAD^+^-triclosan with different molar ratios, pre-incubation times and reservoir solutions. Unfortunately, we failed to observe electron densities for NAD^+^ or triclosan at the expected binding sites in any of the crystal structures of the supposed complexes. This could be explained by the fact that NAD^+^ binds weakly to xoFabV according to isothermal titration calorimetry assay (data not shown) and that triclosan is a rapid, reversible inhibitor of the FabV enzyme [12]. We also attempted to co-crystallise the wild-type xoFabV in complex with NADH but were not able to obtain the complex structure.

As mentioned above, the structures of the potential key residues of xoFabV and ecFabI are similar, which indicates that these residues in xoFabV might play a role in reductase activity in a similar manner to those in ecFabI. To investigate the roles of these residues in FabV enzymatic activity, we expressed and purified the following mutants: S50A, Y53A, Y53F, D111A, F113A, Y226F, Y236A, Y236F, K245A, K245R, V246A and T276A. Because the N-terminal His tag does not dramatically affect the activity [12], the tag was not removed by thrombin cleavage. The gel filtration chromatography curves of all of the mutants were the same as that of the wild-type xoFabV enzyme, indicating that any variations in enzymatic activity resulting from mutagenesis were not caused by conformational changes.

**Figure 6:**
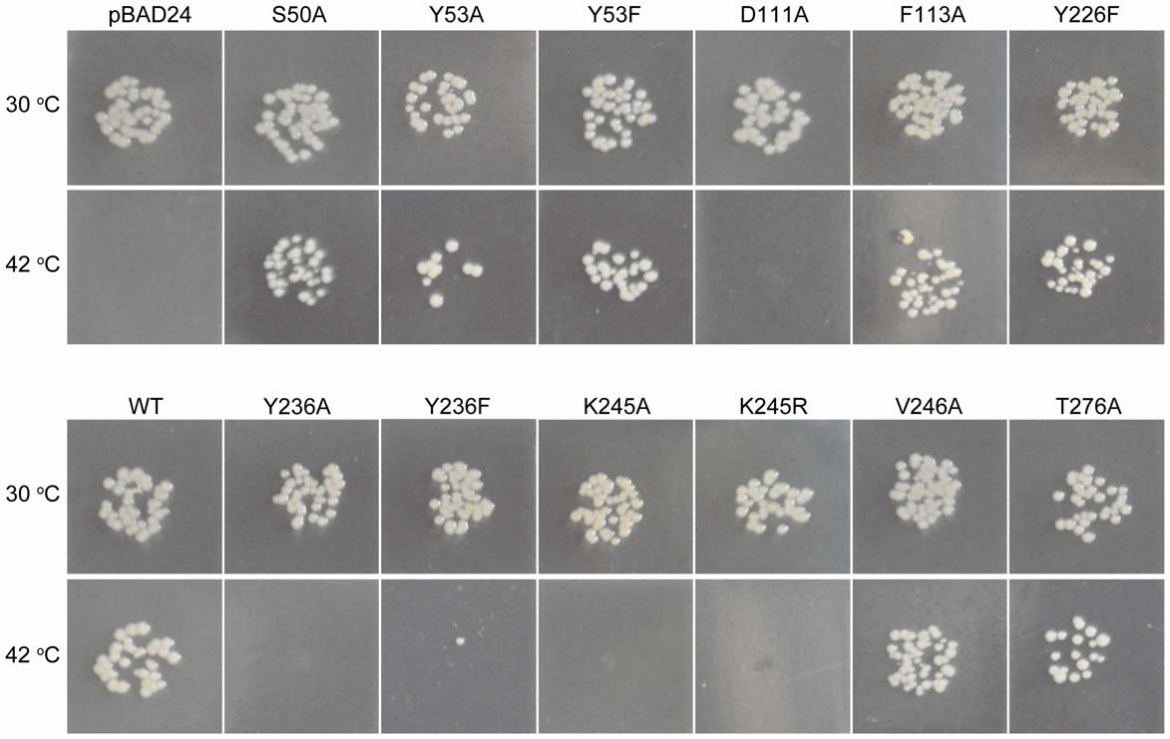
*In vivo* plasmid complementation test. The functions of the wild-type and mutant *xoFabV* genes were validated in the *E. coli fabI* (Ts) strain JP1111. Growth conditions at 30°C are before induction with arabinose; growth conditions at 42°C are after induction with arabinose.

**Figure 7:**
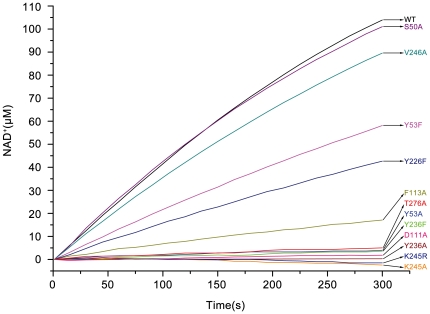
Progress curve analysis of the wild-type and mutant xoFabV variants in the NADH oxidation assay. The enzyme activity of wild-type and mutant xoFabV was determined by monitoring the oxidation of NADH to NAD^+^ at 340 nm. The reaction was initiated by adding the substrate crotonyl-CoA and was monitored for 10 min at 25°C.

The expression of xoFabV mutants D111A, Y236A, Y236F, K245A and K245R in the *E. coli fabI* (Ts) strain JP1111 [2] did not restore *in vivo* fatty acid synthesis at the non-permissive temperature of 42°C (Fig. 6). Mutant Y53A partly restored fatty acid synthesis, while S50A, Y53F, F113A, Y226F, V246A and T276A restored synthesis to the wild-type level. NADH oxidation was assayed *in vitro* with the model substrate crotonyl-CoA. Kinetic parameters were measured only for wild-type xoFabV. The values of *k*_cat_, *K*_m,NADH_ and *K*_m,Crotonyl-CoA_ were 1335±145 min^−1^, 18.7±2.0 µM and 293±16.5 µM, respectively (Table 3), indicating that xoFabV has a similar catalytic efficiency for crotonyl-CoA compared to bmFabV [12]. For the xoFabV mutants, only progress curves were measured (Fig. 7). The curves showed that the enzymatic activities of mutants Y53A, D111A, Y236A, Y236F, K245A, K245R and T276A were not detected in the NADH oxidation assay; Y53F and Y226F catalysed the reduction at about 50% efficiency; F113A had a speed that was one sixth that of the wild-type and mutants S50A and V246A were as efficient as the wild-type enzyme.

**Table 3:** Kinetic parameters for the wild-type xoFabV enzyme.

*k*_cat_ (min^−1^)	*K*_m_ (µM)		*k*_cat_/*K*_m_ (µM^−1^ min^−1^)
	NADH	Crotonyl-CoA	Crotonyl-CoA
1335±145	18.7±2.0	293±16.5	4.5±0.6

The results of the *in vivo* plasmid complementation test and the *in vitro* oxidation assay illustrate that D111, Y236 and K245 are the most important residues for reductase activity. All of the mutations introduced into these three residues completely abolished enzyme function. D111 of xoFabV corresponds to D64 in ecFabI, which interacts with NAD^+^ via hydrogen bonding through the oxygen atom of the side chain, and their conformations are similar. Thus, D111 might also interact with the cofactor in the same manner as D64 in ecFabI. Y236 and K245 are the conserved tyrosine and lysine residues in the Y-X_8_-K motif. Although mutagenesis of Y156F in ecFabI causes a modest decrease in enzymatic activity [24], the fact that Y236A and Y236F completely lost their activity suggests that Y236 plays an essential role in the reduction of the substrate and that it functions via its hydroxyl group. This residue might function as its corresponding partner in ecFabI (Y156), which protonates the enoyl-ACP substrate or binds to the inhibitor through a hydrogen bond. Mutagenesis of K245A and K245R both diminish enzymatic activity, indicating that K245 is essential for the interaction with the cofactor NADH, similarly to the role of Y163 in ecFabI, and the side chain length of K245 is critical for binding the cofactor.

Y53, F113 and T276 are also key residues for enzyme function and might interact with NAD^+^ via hydrogen bonds as their corresponding partners in ecFabI (I20, V65 and T194, respectively). The decreased enzyme activities of mutants Y53A and Y53F indicate that Y53 probably functions via its aromatic side chain rather than only by the hydroxyl group. Mutants F113A and T276A restored fatty acid synthesis *in vivo* but had much lower catalytic efficiencies *in vitro* than the wild-type xoFabV, suggesting that they played important roles in reductase activity. Residue Y226 is the second conserved tyrosine located upstream of the Y-X_8_-K motif, and its corresponding residue in ecFabI (Y146) interacts with both the cofactor and inhibitor via hydrophobic contacts. The variation in the *in vitro* enzyme activity assay for the mutant Y226F reveals that this residue is essential.

Residue V246 corresponds to K245 in bmFabV, which is taken as a second conserved lysine located right after the Y-X_8_-K motif. Although K245 in bmFabV has been demonstrated to be involved in the binding of enoyl substrates and probably functions through its side chain [12], V246 in xoFabV is not a key residue in this reaction, which may be due to their different substrate specificities. Residue S50 was predicted to be involved in the NAD(P)H-binding site in the CD-Search analysis against the Conserved Domain Database, but the replacement of this residue with alanine did not abolish its enzymatic activity, indicating that S50 might not participate in the substrate reduction.

As mentioned above, xoFabV has several additional α-helices (α1 and α11 – α13) and β-strands (β5 – β8 and β11) compared with ecFabI. However, the functions of these secondary structures are not known. To explore their possible roles in the reductase reaction, a CD-Search analysis against the Conserved Domain Database was performed. The analysis indicated that xoFabV has a potential FAD-binding site at its C-terminus, which is conserved as FGFxxxxxDY where x is any residue. In xoFabV, the sequence is FGFGRTDVDY (residues 377–386). To test the ability of the wild-type xoFabV enzyme to bind FAD, co-crystallisation and isothermal titration calorimetry (ITC) assays were performed. Although the conditions for co-crystallisation between xoFabV and FAD were optimised, including various molar ratios, pre-incubation times and reservoir solutions, no convincing electron density for the FAD molecule could be observed at the predicted FAD-binding sites in the crystal structures of the supposed complexes. Also, the ITC assay suggested that the interaction between xoFabV and FAD might be weak, and no curve could be fitted according to the titration data. These indicate that the binding of FAD to xoFabV is probably weak; however, the functions of the additional regions need to be further investigated.

### Conclusion

We determined the first crystal structure of the FabV enzyme with the Y-X_8_-K motif and discussed the differences between this motif and the Y-X_6_-K motif in ecFabI. Also, we identified the key residues involved in the reductase activity. Based on the *in vivo* plasmid complementation test and the *in vitro* NADH oxidation assay, D111, Y236 and K245 are found to be the most essential residues for activity, and Y53, F113, Y226 and T276 are also important for catalysing substrate reduction. Structure comparison and the LIGPLOT analysis suggest that i) Y53, D111, F113, K245 and T276 might interact with the NADH cofactor; ii) Y226 might interact with both the cofactor and substrate (or inhibitor) and iii) Y236 might interact with the substrate (or inhibitor). These findings are extremely useful for the development of antibacterial inhibitors specific for the FabV enzyme in the pathogenic bacteria.

## Materials and Methods

### Enzyme expression and purification

The *Xanthomonas oryzae pv. oryzae* KS-1-21 strain [25] was a gift from Prof. Shiping Wang of Huazhong Agricultural University. The entire *xoFabV* gene was amplified by PCR using a single colony of the KS-1-21 strain as the template. Primers designed for cloning are listed in Table 4. The gene was inserted into the pET-28b(+) vector using the 5′ *Nde* I and 3′ *Hin*d III restriction sites, and a sequence encoding a His tag and a thrombin cleavage site was introduced upstream of the *xoFabV* gene. The recombinant SeMet-xoFabV was expressed as described [26] except that the pET-28b(+) vector and *E. coli* BL21(DE3) strain were used. After harvesting the bacteria by centrifugation, the cells were lysed by sonication in equilibration buffer (50 mM Tris-HCl, 500 mM NaCl, 50 mM imidazole, pH 8.0), and the lysate was then applied to Ni^2+^-affinity resin (Chelating Sepharose™ Fast Flow, GE Healthcare, NJ, USA) in a Poly-Prep™ chromatography gravity column (Bio-Rad, CA, USA). After washing the column with equilibration buffer, the target protein was eluted with Tris-HCl buffer containing 200 mM imidazole. The protein was purified using a Superdex™ 200 (10/300 GL) column in gel filtration buffer (50 mM Tris-HCl, 100 mM NaCl, 1 mM DTT, pH 8.0). The xoFabV monomer was collected and concentrated, and its concentration was measured by the Bradford method.

**Table 4:** Primers used for cloning and mutagenesis.

Name	Sequence^a^
xoFabV forward	5′ GACCAGGAGTCCCATATGATCATCCATCCCA 3′
xoFabV reverse	5′ CATCCAAGCTTTCAAGCCAACTCGATGCA 3′
S50A forward	5′ AAAGTACTGGTGATCGGCGCGGCCAGCGGCTA 3′
S50A reverse	5′ C CGCGCCGATCACCAGTACTTTCTTGGGGCCG 3′
Y53A forward	5′ GCGCGTCCAGCGGCGCTGGCCTGGCCTCGC 3′
Y53A reverse	5′ GC GCCGCTGGACGCGCCGATCACCAGTACTT 3′
Y53F forward	5′ CGTCCAGCGGCTTTGGCCTGGCC 3′
Y53F reverse	5′ A AGCCGCTGGACGCGCCGATCAC 3′
D111A forward	5′ TAGCAAGTCGATCAATGGCGCTGCGTTTTCCG 3′
D111A reverse	5′ G CGCCATTGATCGACTTGCTATACAACCCGGCG 3′
F113A forward	5′ ATCAATGGCGATGCGGCTTCCGACGCC 3′
F113A reverse	5′ GC CGCATCGCCATTGATCGACTTGCTAT 3′
Y226A forward	5′ GC TATCGGGACCGAGATCACCTGG 3′
Y226A reverse	5′ TCTCGGTCCCGATAGCGCTGAAAGCCACGC 3′
Y226F forward	5′ GCGTGGCTTTCAGCTTTATCGGGACCG 3′
Y226F reverse	5′ A AGCTGAAAGCCACGCTGCGCGC 3′
Y236A forward	5′ CGAGATCACCTGGCCGATCGCTTGGCATGG 3′
Y236A reverse	5′ GC GATCGGCCAGGTGATCTCGGTCCCGAT 3′
Y236F forward	5′ TCACCTGGCCGATCTTTTGGCATGGC 3′
Y236F reverse	5′ A AGATCGGCCAGGTGATCTCGGTCC 3′
K245A forward	5′ GGCGCACTCGGCAAGGCCGCGGTCGATCTT 3′
K245A reverse	5′ GC GGCCTTGCCGAGTGCGCCATGCCAATAG 3′
K245R forward	5′ CACTCGGCAAGGCCAGGGTCGATCTT 3′
K245R reverse	5′ C TGGCCTTGCCGAGTGCGCCATGCCA 3′
V246A forward	5′ CGCACTCGGCAAGGCCAAGGCCGATCTTGAC 3′
V246A reverse	5′ G CCTTGGCCTTGCCGAGTGCGCCATGC 3′
T276A forward	5′ GGTGCTCAAGTCGGTGGTCGCCCAGGCCAG 3′
T276A reverse	5′ C GACCACCGACTTGAGCACCGCCACATTGG 3′

a Restriction sites and mutated sites are underlined.

The xoFabV mutants S50A, Y53A, Y53F, D111A, F113A, Y226F, Y236A, Y236F, K245A, K245R, V246A and T276A were constructed using the Easy Mutagenesis Kit (TransGen, Beijing, China). Primers designed for mutagenesis are listed in Table 4. The wild-type and mutant xoFabV enzymes were expressed in BL21(DE3) cells at 16°C, and expression was induced by the addition of 0.2 mM IPTG for 20 h. The purification procedure was the same as above, but no DTT was added.

### Crystallisation, data collection and structure determination

SeMet-xoFabV protein crystals were grown using the hanging-drop vapour diffusion method in a reservoir solution of 100 mM Bis-Tris (pH 6.5) containing 200 mM NaCl, 1 mM DTT and 15% PEG 3350. Crystals appeared in 24 h and were of high quality. Two diffraction datasets were collected. The HKL-2000 package [27] was used for data processing. The crystal belonged to space group *P*2_1_2_1_2_1_, with cell dimensions of a = 50.52 Å, b = 74.53 Å and c = 107.39 Å. PHENIX [28] was used for two-wavelength MAD phasing and automatic model building. One xoFabV monomer was found in the asymmetric unit, and five out of the six expected selenium atoms were located in each monomer. Several iterative rounds of manual building in COOT and refinement in PHENIX were performed until acceptable *R*_work_ (18.5%) and *R*_free_ (20.5%) values were achieved. The stereochemical quality of the final model at 1.6 Å was validated using PROCHECK [29], which showed that 93.2% of the residues are within the most favoured region of the Ramachandran plot and 6.8% are within the additional allowed region. The Matthews coefficient [30] was 2.1 Å^3^ Da^−1^, and the solvent content was 44.2%.

### Plasmid complementation test and NADH oxidation assay

To test their abilities to restore *in vivo* fatty acid synthesis, wild-type and mutant *xoFabV* genes were cloned into the arabinose-inducible pBAD24 vector [31], [32] and transformed into the *fabI* (Ts) strain JP1111. The cultures were grown at 30°C, induced with 0.02% arabinose and then shifted to 42°C.

The enzyme activities of the wild-type and mutant xoFabVs were determined by monitoring the oxidation of NADH to NAD^+^ at 340 nm using an NADH extinction coefficient of 6220 M^−1^ [33]. Each 150 µL of reaction mixture contained 75 nM purified His-tagged xoFabV, 200 µM NADH, 200 µM crotonyl-CoA and 1 mM NaCl in 20 mM Tris-HCl buffer (pH 7.5). The reaction was initiated by adding the substrate crotonyl-CoA and was monitored for 10min at 25°C. The kinetic constants of the wild-type xoFabV enzyme were analysed using Microsoft Excel 2003. Progress curves of the mutant xoFabV enzymes were constructed using Origin Pro version 8.0.

### ITC assay and co-crystallisation of xoFabV with FAD

The isothermal titration calorimetry assay was performed at 25°C using an iTC_200_ Microcal calorimeter (Microcal, NJ, USA). FAD and wild-type xoFabV were both dissolved in ITC buffer (50 mM Tris-HCl, 100 mM NaCl, pH 8.0). Sixty microliters of 4 mM FAD solution was loaded into the syringe as the ligand, and 250 µL of 200 µM xoFabV was injected into the cell as the binding partner. The ligand was injected into the protein in 20 drops with 0.5 µL used for the first drop and 2 µL used for the remaining 19 drops. A stirring speed of 2000 rpm and a time interval of 120 s between drops were set to ensure that every drop of ligand solution was completely mixed with the binding partner. The ligand was titrated into protein-free ITC buffer as a reference, and the data were analysed using Origin software version 7.0.

Wild-type xoFabV and FAD were mixed at 1∶2 to 1∶10 molar ratios and incubated at 4°C for 1 h prior to being co-crystallised under the same conditions as SeMet-xoFabV but without adding DTT. The diffraction datasets of the crystals were collected using a VariMax™ and MicroMax™-007 X-ray generator (Rigaku) at the Institute of Biophysics, Chinese Academy of Sciences. The structures were determined with PHENIX by the molecular replacement method using the SeMet-xoFabV structure as the search model.
